# Esophageal Perforation and Fistulization After Ingestion of Multiple Foreign Bodies: A Case Report

**DOI:** 10.7759/cureus.101036

**Published:** 2026-01-07

**Authors:** Kamily Sara Turt, Beatriz Lanza Pauli, Gabriel Mondin Nogueira, Hector Sbaraini Fontes, Rebeca Trevisan Iurkiewiecz

**Affiliations:** 1 General Surgery, Complexo Hospitalar do Trabalhador, Curitiba, BRA; 2 General Surgery, Complexo do Hospital de Clínicas da Universidade Federal do Paraná (UFPR), Curitiba, BRA

**Keywords:** esophageal fistula, esophageal perforation, esophagorrhaphy, foreign body ingestion, trauma critical care

## Abstract

Esophageal perforation is a life-threatening condition associated with high morbidity and mortality, particularly when diagnosis or treatment is delayed. Foreign body ingestion is a known cause of esophageal injury and may result in severe complications requiring surgical intervention. We report the clinical course and therapeutic management of a young adult who developed cervical esophageal perforation after deliberate ingestion of multiple foreign bodies associated with acute cocaine intoxication and self-induced emesis. The patient presented with cervical pain, extensive subcutaneous emphysema, and pneumomediastinum and underwent exploratory cervicotomy with primary esophagorrhaphy. The postoperative course was complicated by hemopneumothorax requiring thoracoscopic drainage and subsequent esophageal fistulization, which was successfully managed conservatively. This case highlights the diagnostic challenges, multidisciplinary decision-making, and potential complications associated with esophageal perforation secondary to deliberate foreign body ingestion in high-risk patients.

## Introduction

Esophageal perforation is a rare but life-threatening condition associated with high morbidity and mortality, mainly due to the rapid progression to mediastinitis, sepsis, and respiratory failure following leakage of esophageal contents into adjacent anatomical compartments. Despite advances in diagnostic imaging and surgical techniques, delayed recognition and inadequate source control remain the main determinants of poor outcomes in these patients [1,2].

Foreign body ingestion represents an important etiology of esophageal injury in adults and differs significantly from pediatric cases, as it is frequently intentional and associated with psychiatric disorders or substance abuse. Although most ingested foreign bodies pass spontaneously through the gastrointestinal tract, ingestion of multiple, irregular, or sharp objects markedly increases the risk of impaction, perforation, and need for invasive intervention [3,4]. Most published reports, however, describe accidental ingestion of a single object or isolated risk factors.

Reports of esophageal perforation resulting from the deliberate ingestion of multiple heterogeneous foreign bodies in the setting of acute cocaine intoxication are scarce, particularly when complicated by extensive cervical involvement, pneumomediastinum, and subsequent fistulization. This combination represents a uniquely high-risk clinical scenario that poses significant diagnostic and therapeutic challenges, requiring early imaging, multidisciplinary evaluation, and individualized surgical decision-making.

This case report describes the clinical course and therapeutic management of a patient who developed cervical esophageal perforation after the deliberate ingestion of multiple foreign bodies associated with cocaine intoxication, complicated by subcutaneous emphysema and pneumomediastinum, and who underwent surgical esophagorrhaphy with subsequent fistula formation. The report aims to highlight critical aspects of clinical reasoning, surgical management, and multidisciplinary care in this uncommon and complex presentation.

## Case presentation

A 23-year-old previously healthy male presented to the emergency department with cervical pain after cocaine intoxication, manual induction of emesis, and deliberately ingesting multiple foreign bodies, including an ashtray containing cigarette butts and ashes, a climbing carabiner, two keys, a keychain, a toothpick, and a coin. He was transported by prehospital emergency medical services to the emergency department. On initial evaluation, he was breathing comfortably on room air. He exhibited sinus tachycardia at presentation, likely related to pain and acute cocaine intoxication, while remaining hemodynamically stable, complaining of neck pain and swelling, as well as oral lesions without active bleeding. He denied other complaints and was immediately monitored, with peripheral venous access obtained in the right upper limb.

On arrival at the emergency department, his blood pressure was 138/85 mmHg, heart rate 117 beats per minute, and oxygen saturation 95% on room air. He appeared agitated, with a Glasgow Coma Scale (GCS) score of 15, with isocoric and reactive pupils. The airway was patent, with oral lesions, and the patient presented extensive subcutaneous emphysema throughout the anterior cervical region, crepitant on palpation. Pulmonary examination revealed eupnea with symmetric chest expansion, without pain on palpation of the costal arches or sternum. He remained hemodynamically stable with good peripheral perfusion. Cardiac auscultation revealed normal and regular heart sounds. A focused assessment with sonography for trauma (FAST) performed in the emergency department was negative in all evaluated windows. The patient was kept nil per os.

**Figure 1:**
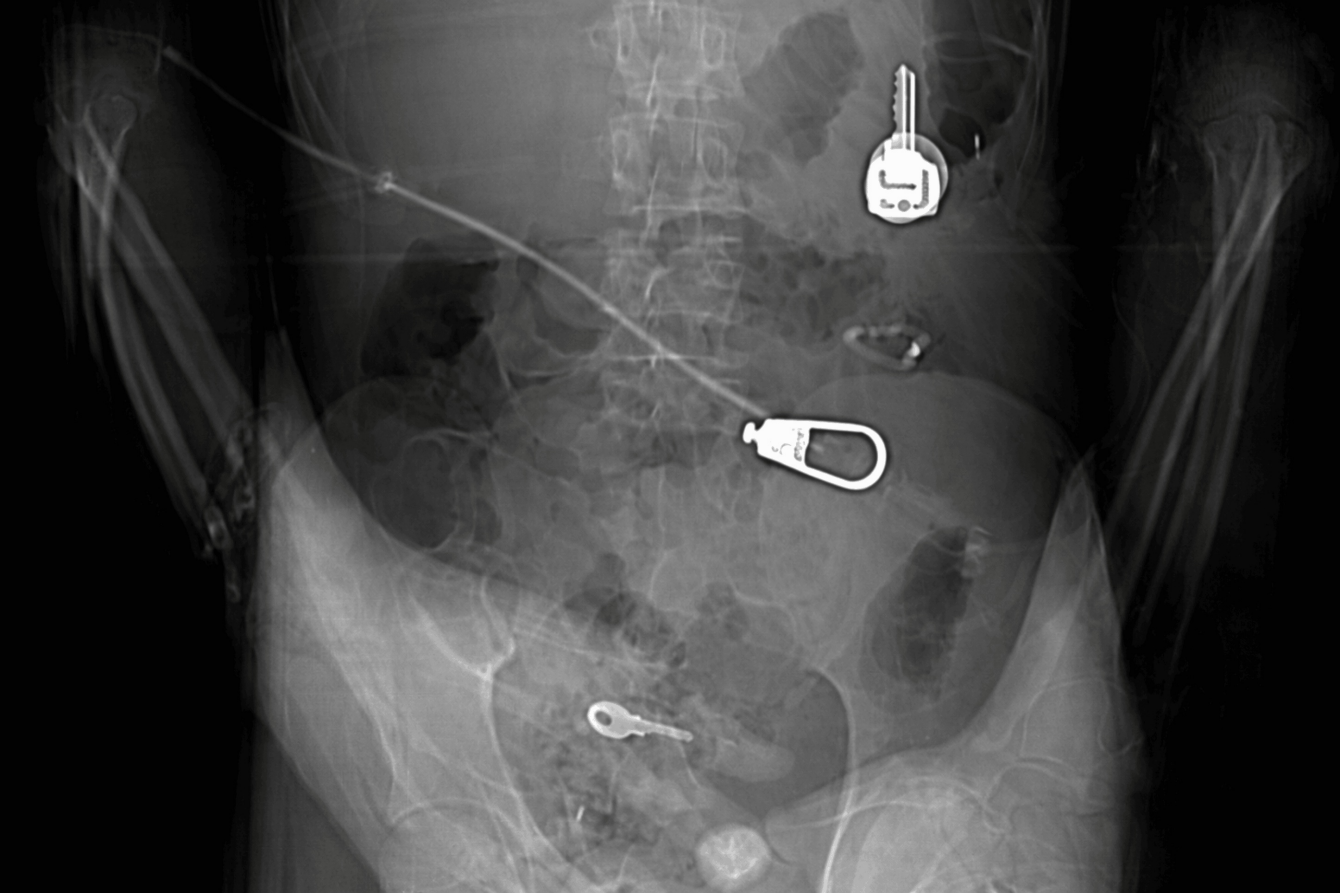
Anteroposterior CT scout image showing multiple radiopaque foreign bodies throughout the gastrointestinal tract.

**Figure 2:**
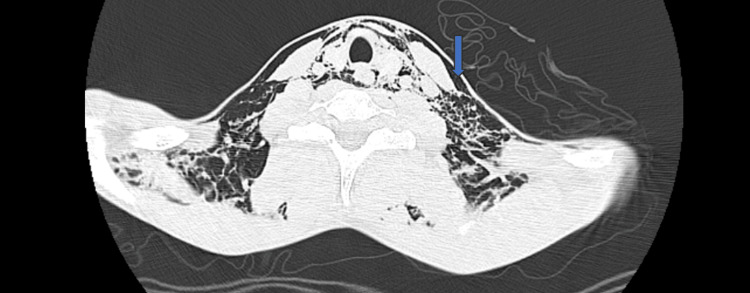
Axial chest CT image showing bilateral cervical subcutaneous emphysema. The arrow highlights air dissecting the subcutaneous tissues.

**Figure 3:**
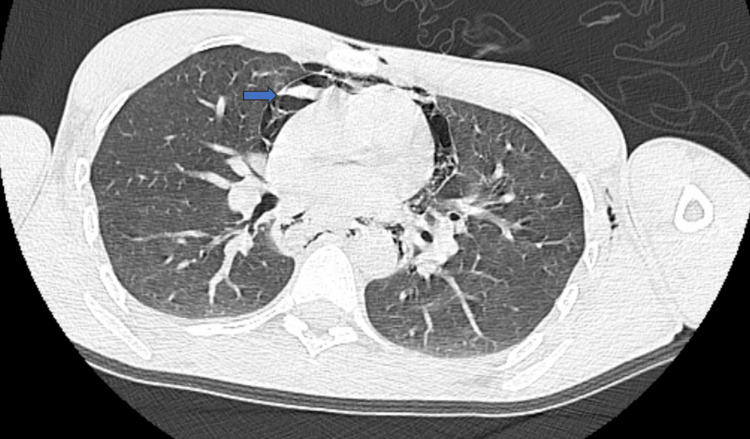
Axial chest CT image showing pneumomediastinum. The arrow highlights free mediastinal air.

He was referred for imaging studies. Contrast-enhanced computed tomography (CT) demonstrated extensive subcutaneous emphysema in the cervical and thoracic regions, with marked pneumomediastinum. There was no evidence of hemopneumothorax, free abdominal fluid, or pneumoperitoneum. Multiple foreign bodies were identified throughout the gastrointestinal tract, from the stomach to the rectal region (Figures 1-3). Cervical CT with oral contrast revealed signs of high esophageal perforation, evidenced by extensive contrast extravasation along the entire esophageal length, making precise localization of the perforation impossible.

The patient was taken to the operating room for upper gastrointestinal endoscopy to define the size and location of the laceration. Endoscopic examination revealed a laceration in the left piriform recess extending below the cricopharyngeal muscle constriction, with food debris present. The middle and distal esophagus showed no evident lesions. The stomach contained multiple foreign bodies, including plastic and paper, without gross mucosal injury. The duodenum also contained multiple foreign bodies. A nasoenteral feeding tube was placed.

The patient was under the care of the surgical team, which presented the clinical scenario for multidisciplinary case review to determine the most appropriate management strategy given its complexity and rarity. Following multidisciplinary consultation with the endoscopy, radiology, and thoracic surgery teams, exploratory cervicotomy with primary esophagorrhaphy was indicated for confirmed esophageal perforation. An anterior cervical dissection was performed to identify the esophagus, revealing an approximately 2 cm laceration of the cervical esophagus at the level of the thyroid cartilage, with a large amount of periesophageal foreign material. The lesion was repaired with simple interrupted sutures. Two drains were placed in the left cervical space and exteriorized through the incision. Broad-spectrum antibiotic therapy was initiated, and the patient was transferred to the intensive care unit (ICU) for clinical management and surveillance for sepsis due to the high risk of mediastinitis.

**Figure 4:**
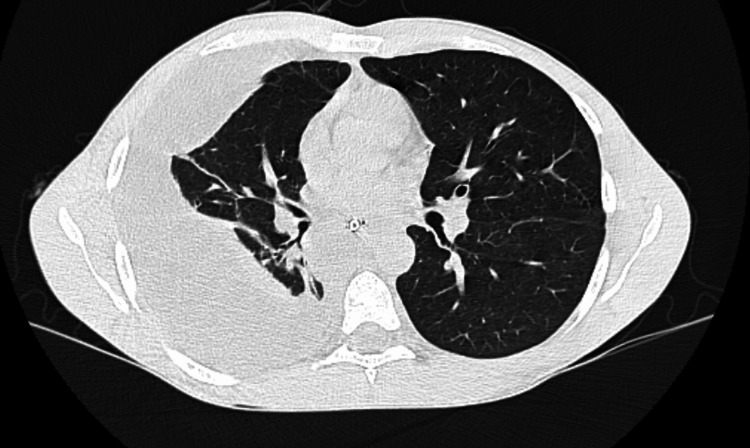
Follow-up axial chest CT image demonstrating complete resolution of the pneumomediastinum, with associated right-sided pleural effusion.

Eight days after surgery, the patient developed hemopneumothorax (Figure 4) and underwent video-assisted thoracoscopic surgery with closed pleural drainage and chest tube placement connected to a water-seal system, with resolution of the complication.

Three weeks after surgery, the patient developed an esophageal fistula arising from the mid-portion of the esophagorrhaphy, with extension into the periesophageal soft tissues at the T2 vertebral level, evidenced by an oral methylene blue test with extravasation through the surgical drain placed during cervicotomy. The patient remained in good general condition without additional complications. Exclusive enteral nutrition via nasoenteral tube was maintained due to the high risk of mediastinitis and sepsis, and atropine was prescribed to reduce salivary secretion. Conservative measures were adopted for the treatment of the esophageal fistula.

Throughout hospitalization, the patient was followed by the psychiatry team for management of psychiatric conditions associated with cocaine use and ingestion of multiple foreign bodies, as well as abstinence during hospitalization. Pharmacological treatment and psychiatric supportive care were provided. Multidisciplinary follow-up was also conducted by nutrition, physiotherapy, psychology, and nursing teams.

The patient remained hospitalized with a favorable clinical evolution. Thirteen days after fistula diagnosis, a repeat oral methylene blue test showed no extravasation, indicating fistula resolution. The surgical team opted for drain removal and planned hospital discharge after a new endoscopic evaluation. Outpatient follow-up was scheduled due to satisfactory clinical recovery. The patient was referred for continued outpatient psychiatric care.

## Discussion

Esophageal perforation is one of the most serious injuries of the digestive tract in terms of morbidity and mortality, potentially leading to severe mediastinitis and sepsis due to leakage of digestive secretions and food particles [1]. Esophageal perforation may result from different factors, including direct injury caused by blunt or penetrating trauma [5]. In the present case, perforation occurred through a complex mechanism involving substance abuse, forceful self-induced emesis, and ingestion of multiple foreign bodies, resulting in extensive contamination of cervical and mediastinal spaces.

In cases of foreign body ingestion, accurate characterization of the ingested object and a careful clinical history are essential for appropriate diagnostic and therapeutic guidance. Approximately 80% to 90% of ingested foreign bodies pass spontaneously through the gastrointestinal tract, with or without symptoms; however, some require intervention. Potential complications related to foreign body impaction or transit include the need to recognize emergency situations and apply the most appropriate management strategies. An increased rate of ingestion of foreign bodies is also associated with individuals with psychiatric disorders and with alcohol or drug use [3,6,7]. In this patient, the ingestion of multiple heterogeneous foreign bodies following cocaine use represented a high-risk scenario, reinforcing the importance of early imaging and vigilant assessment for esophageal injury even in initially stable patients.

Regarding esophageal injuries, current treatment recommendations depend on the time interval between the event and diagnosis, the patient’s clinical condition, and the location of the injury (cervical, thoracic, or abdominal esophagus). Cervical lesions are typically managed with lateral cervicotomy, debridement of devitalized tissue, layered closure, local drainage, and broad-spectrum antibiotic therapy [8]. 

Esophageal perforation is associated with high morbidity and mortality due to the rapid development of mediastinitis, sepsis, and respiratory failure resulting from leakage of esophageal contents into critical anatomical compartments. Early clinical suspicion is essential and should be raised by findings such as severe cervical or thoracic pain, fever, tachycardia, subcutaneous emphysema, cervical crepitus, dyspnea, pleural effusion, pneumomediastinum, or disproportionate pain after endoscopic manipulation. Multiple studies demonstrate that time to diagnosis and prompt contamination control are the main prognostic determinants, with delayed recognition leading to significantly worse outcomes. Accordingly, the World Society of Emergency Surgery (WSES) classifies esophageal perforation as a true surgical emergency and recommends management based on three key pillars: time since injury, degree of contamination, and clinical stability. Contrast-enhanced computed tomography of the neck and chest is the imaging modality of choice to assess lesion extent and complications. While conservative management may be considered in carefully selected, clinically stable patients with contained perforations, strict selection criteria are mandatory to avoid clinical deterioration and increased mortality [2].

In a large retrospective series involving 275 adults with foreign body ingestion, it was demonstrated that although most cases can be managed endoscopically, a clinically relevant proportion evolved with severe complications, including esophageal perforation identified in 20 patients. Factors such as ingestion of multiple foreign bodies, irregular or sharp objects, and delayed intervention were associated with a higher risk of transmural injury and need for invasive treatment. These findings reinforce that foreign body ingestion in adults should not be regarded as a benign event, particularly when multiple objects are involved, requiring high suspicion for perforation, early imaging, and rapid therapeutic decision-making [9]. 

In adults, the risk of esophageal perforation is directly related to the type of foreign body ingested, being significantly higher with sharp or pointed objects such as bones, toothpicks, and dental prostheses, as well as button batteries and magnets, which may cause chemical injury and pressure necrosis in addition to mechanical damage. Given this high potential for injury, the European Society of Gastrointestinal Endoscopy (ESGE) recommends maximum priority for endoscopic removal of such objects. The guideline emphasizes that endoscopy timing should be risk-based rather than indiscriminate: situations such as complete esophageal obstruction, sharp objects, or batteries in the esophagus require emergent intervention (ideally within two hours and no later than six hours), whereas other esophageal foreign bodies without complete obstruction or food bolus impaction without signs of complication may be managed urgently within 24 hours, an approach shown to reduce complications in large clinical series [10]. In the present case, endoscopy was essential for lesion characterization; however, nonoperative management and exclusive endoscopic therapies were considered inappropriate due to the extent of mediastinal contamination and tissue injury. Endoscopic closure or isolated stent placement would not have adequately addressed the established contamination, whereas delayed or drainage-only strategies would have carried an unacceptably high risk of persistent infection and fistula formation. Likewise, esophagectomy was not indicated, given the absence of extensive necrosis or underlying esophageal disease. Therefore, exploratory cervicotomy with primary esophagorrhaphy was selected as the most appropriate approach, allowing definitive source control, direct repair of viable tissue, and effective drainage, in accordance with current surgical recommendations.

Deliberate foreign body ingestion in adults is strongly associated with psychiatric disorders, frequently coexisting with substance abuse, which contributes to impulsive behavior and recurrence of ingestion episodes. In a multicenter retrospective study, most cases occurred in patients with underlying mental illness, particularly personality disorders, and a significant proportion had comorbid alcohol or illicit drug use. These patients often required multiple endoscopic interventions, reinforcing that foreign body ingestion in this context should be understood as part of a complex behavioral pattern requiring multidisciplinary management and psychiatric follow-up to prevent recurrence and complications [4]. Accordingly, psychiatric evaluation and continued outpatient follow-up were essential components of care in this patient, aiming to address substance abuse and reduce the risk of recurrence.

From an educational perspective, this case emphasizes that esophageal perforation should be actively ruled out in adult patients presenting with cervical pain and subcutaneous emphysema after foreign body ingestion, especially in the setting of substance use or psychiatric comorbidity. Early contrast-enhanced CT and multidisciplinary discussion are critical to guide timely source control and prevent catastrophic complications. Additionally, long-term psychiatric follow-up is essential to reduce recurrence and healthcare utilization.

Reports of esophageal perforation secondary to foreign body ingestion in adults are well described in the literature; however, most published cases involve a single ingested object, accidental ingestion, or isolated risk factors. In contrast, the present case is distinguished by the deliberate ingestion of multiple heterogeneous foreign bodies in the setting of acute cocaine intoxication and forceful self-induced emesis, resulting in cervical contamination and subsequent fistulization despite timely surgical management. The coexistence of substance abuse, psychiatric vulnerability, ingestion of multiple sharp and irregular objects, and progression to postoperative fistula formation represents an uncommon and particularly high-risk clinical scenario that is rarely reported in combination. This case, therefore, adds meaningful insight to the existing literature by highlighting a unique pathophysiological constellation and underscores the importance of early imaging, multidisciplinary decision-making, and integrated surgical and psychiatric management in similarly complex presentations.

## Conclusions

This case illustrates a severe and uncommon presentation of cervical esophageal perforation resulting from deliberate ingestion of multiple foreign bodies in a young adult, associated with cocaine use and self-induced emesis, highlighting the complex interaction between clinical, behavioral, and psychiatric factors. As described in the literature, the type of foreign body ingested, diagnosis time, and effective control of contamination are central determinants of prognosis, which was reflected in the need for early surgical intervention, intensive management, and strict surveillance for complications such as mediastinitis and esophageal fistula. The patient’s favorable outcome underscores the importance of an integrated multidisciplinary strategy involving endoscopy, surgery, intensive care, and psychiatric follow-up, not only for treatment of the esophageal injury but also for prevention of recurrence. Thus, this report reinforces that, in adults, foreign body ingestion should be regarded as a potentially lethal event, frequently associated with psychiatric disorders and substance use, requiring early recognition and a comprehensive, patient-centered approach.
